# Maximal distant entanglement in Kitaev tube

**DOI:** 10.1038/s41598-018-29691-1

**Published:** 2018-08-15

**Authors:** P. Wang, S. Lin, G. Zhang, Z. Song

**Affiliations:** 1 School of Physics, Nankai University, Tianjin, 300071 China; 2 College of Physics and Materials Science, Tianjin Normal University, Tianjin, 300387 China

## Abstract

We study the Kitaev model on a finite-size square lattice with periodic boundary conditions in one direction and open boundary conditions in the other. Based on the fact that the Majorana representation of Kitaev model is equivalent to a brick wall model under the condition *t* = Δ = μ, this system is shown to support perfect Majorana bound states which is in strong localization limit. By introducing edge-mode fermionic operator and pseudo-spin representation, we find that such edge modes are always associated with maximal entanglement between two edges of the tube, which is independent of the size of the system.

## Introduction

Topological materials have become the focus of intense research in the last years^1–4^, since they not only exhibit new physical phenomena with potential technological applications, but also provide a fertile ground for the discovery of fermionic particles and phenomena predicted in high-energy physics, including Majorana^5–10^, Dirac^11–17^ and Weyl fermions^18–26^. These concepts relate to Majorana edge modes. A gapful phase can be topologically non-trivial, commonly referred to as topological insulators and superconductors, the band structure of which is characterized by nontrivial topology. The number of Majorana edge modes is determined by bulk topological invariant. In general, edge states are the eigenstates of Hamiltonian that are exponentially localized at the boundary of the system. A particularly important concept is the bulk-edge correspondence, which links the nontrivial topological invariant in the bulk to the localized edge modes. On the other hand, Majorana edge modes have been actively pursued in condensed matter physics^27–33^ since spatially separated Majorana fermions lead to degenerate ground states, which encode qubits immune to local dechoerence^34^. There have been theoretical proposals for detecting Majorana fermions in 2D semiconductor heterostructures^35,36^, topological insulator-superconductor proximity^5,37–40^, 1D spin-orbit-coupled quantum wires^6,9,41–48^ and cold atom systems^33,49–53^. Experimentally, it is claimed that indirect signatures of Majorana fermions in topological superconductors have been observed^7,8,54–62^. So far, the thoretically predicted Majorana bound state in literatures requires the system in thermodynamic limit. An interesting question is whether there exists the Majorana bound state in a small sized system, or the topological feature is a prerequisite for Majorana bound state. The existence of such a type of edge mode would indicate that the bulk topology is not necessary to the spatially separated Majorana fermions and may provide an alternative way to detect and utilize Majorana fermions.

In this paper, we study the Majorana edge modes in the Kitaev model on a square lattice based on analytical solutions. In contrast to previous studies based on open boundary conditions in two directions, we focus on a finite-length cylindrical lattice. We show that the Majorana representation of Kitaev model is related to a brick wall model, based on which this model in a finite-length cylindrical geometry supports the perfect Majorana bound states under the condition $t={\rm{\Delta }}=\mu$. The perfect Majorana bound state is in the strong localization limit. This Majorana zero mode has two notable features: (i) The edge-mode states exhibit maximal entanglement between the two edges of the cylinder; (ii) By introducing edge-mode pesudospin operators, we find that the edge mode relates to a conserved observable. Remarkably, the expectation values of two types of pseudospins for eigenstates indicate the coexistence of both bosonic and fermionic excitations. And the eigenstates also possess maximal entanglement about the bosonic and fermionic modes. These results provide a way to detect the Majorana bound states in *p*-wave superconductors.

## Model

We consider the Kitaev model on a square lattice which is employed to depict 2D *p*-wave superconductors. The Hamiltonian of the tight-binding model on a square lattice takes the following forma

1 $$\begin{array}{rcl}H & = & -t\sum _{{\bf{r}}{\boldsymbol{,}}{\bf{a}}}{c}_{{\bf{r}}}^{\dagger }{c}_{{\bf{r}}+{\bf{a}}}-{\rm{\Delta }}\sum _{{\bf{r}},{\bf{a}}}{c}_{{\bf{r}}}{c}_{{\bf{r}}+{\bf{a}}}+h.\,c.\\  &  & +\mu \sum _{{\bf{r}}}(2{c}_{{\bf{r}}}^{\dagger }{c}_{{\bf{r}}}-1),\end{array}$$

where **r** is the coordinates of lattice sites and *c*_**r**_ is the fermion annihilation operators at site **r**. Vectors **a** = *a***i**, *a***j**, are the lattice vectors in the *x* and *y* directions with unitary vectors **i** and **j**. The hopping between (pair operator of) neighboring sites is described by the hopping amplitude *t* (the real order parameter Δ). The last term gives the chemical potential. Imposing boundary conditions on both directions, the Hamiltonian can be exactly diagonalized. The Kitaev model on a honeycomb lattice and chain provides well-known examples of systems with such a bulk-boundary correspondence^63–69^. It is well known that a sufficient long chain has Majorana modes at its two ends^70^. A number of experimental realizations of such models have found evidence for such Majorana modes^7,54,56,57,71^. In contrast to previous studies based on system in thermodynamic limit, we focus on the Kitaev model on a finite lattice system. This is motivated by the desire to get a clear physical picture of the egde mode via the investigation of a small system. We first study the present model from the description in terms of Majorana fermions.

We introduce Majorana fermion operators

2 $${a}_{{\bf{r}}}={c}_{{\bf{r}}}^{\dagger }+{c}_{{\bf{r}}},{b}_{{\bf{r}}}=-\,i({c}_{{\bf{r}}}^{\dagger }-{c}_{{\bf{r}}}),$$

which satisfy the relations

3 $$\begin{array}{rcl}\{{a}_{{\bf{r}}},{a}_{{\bf{r}}^{\prime} }\} & = & 2{\delta }_{{\bf{r}},{\bf{r}}^{\prime} },\{{b}_{{\bf{r}}},{b}_{{\bf{r}}^{\prime} }\}=2{\delta }_{{\bf{r}},{\bf{r}}^{\prime} },\\ \{{a}_{{\bf{r}}},{b}_{{\bf{r}}^{\prime} }\} & = & 0,{a}_{{\bf{r}}}^{2}={b}_{{\bf{r}}}^{2}=1.\end{array}$$

Then the Majorana representation of the Hamiltonian is

4 $$\begin{array}{rcl}H & = & -\frac{1}{4}\sum _{{\bf{r}}}[i(t+{\rm{\Delta }})\sum _{{\bf{a}}}{a}_{{\bf{r}}}{b}_{{\bf{r}}+{\bf{a}}}\\  &  & +i(t-{\rm{\Delta }})\sum _{{\bf{a}}}{b}_{{\bf{r}}}{a}_{{\bf{r}}+{\bf{a}}}+i2\mu {a}_{{\bf{r}}}{b}_{{\bf{r}}}+h.\,c\mathrm{.]}.\end{array}$$

It represents a dimerized brick wall lattice (or honeycomb lattice) with extra hopping term *b*_**r**_*a*_**r**+**a**_.

## Majorana Edge Modes

Let us consider a simple case to show that Majorana modes can appear on some edges. Taking *t* = Δ = *μ* the Hamiltonian reduces to

5 $${H}_{BW}=-\,\frac{t}{2}\sum _{{\bf{r}}}(i{a}_{{\bf{r}}}\sum _{{\bf{a}}}{b}_{{\bf{r}}+{\bf{a}}}+i{a}_{{\bf{r}}}{b}_{{\bf{r}}}+h.\,c.).$$

which corresponds to the original Kitaev model

6 $$\begin{array}{rcl}{H}_{BW} & = & -t\sum _{{\bf{r}}{\boldsymbol{,}}{\bf{a}}}({c}_{{\bf{r}}}^{\dagger }{c}_{{\bf{r}}+{\bf{a}}}+{c}_{{\bf{r}}}{c}_{{\bf{r}}+{\bf{a}}})+h.\,c.\\  &  & +t\sum _{{\bf{r}}}(2{c}_{{\bf{r}}}^{\dagger }{c}_{{\bf{r}}}-1).\end{array}$$

Now, we consider a finite lattice system on a cylindrical geometry by taking the periodic boundary condition in one direction and open boundary in another direction. For a *M* × *N* Kitaev model, the Majorana Hamiltonian can be explicitly expressed as

7 $$\begin{array}{rcl}{H}_{BW} & = & -\frac{it}{2}\sum _{m=1}^{M}\sum _{n=1}^{N}({a}_{m,n}{b}_{m,n}+{b}_{m+\mathrm{1,}n}{a}_{m,n}\\  &  & +{b}_{m,n+1}{a}_{m,n}-h.\,c\mathrm{.),}\end{array}$$

by taking ${\bf{r}}=m{\bf{i}}+n{\bf{j}}\to (m,n)$. The boundary conditions are ${b}_{m\mathrm{,1}}={b}_{m,N+1},{a}_{M+\mathrm{1,}n}=\mathrm{0,}\,{b}_{M+\mathrm{1,}n}=0$.

Consider the Fourier transformations of Majorana operators

8 $${a}_{m,n}=\frac{1}{\sqrt{N}}\sum _{K}{e}^{-iKn}{a}_{m,K},$$

9 $${b}_{m,n}=\frac{1}{\sqrt{N}}\sum _{K}{e}^{-iKn}{b}_{m,K},$$

where the wave vector *K* = 2*πl*/*N*, *l* = 1, …, *N*. Here *a*_*m,K*_ and *b*_*m,K*_ represent the linear combinations of Majorana fermion operator. These are not standard Majorana fermions since

10 $${a}_{m,K}^{\dagger }={a}_{m,-K},{b}_{m,K}^{\dagger }={b}_{m,-K},$$

except the case with *K* = 0, where

11 $${a}_{m\mathrm{,0}}^{\dagger }={a}_{m\mathrm{,0}}=\frac{1}{\sqrt{N}}\sum _{n=1}^{N}{a}_{m,n},$$

12 $${b}_{m\mathrm{,0}}^{\dagger }={b}_{m\mathrm{,0}}=\frac{1}{\sqrt{N}}\sum _{n\mathrm{=1}}^{N}{b}_{m,n},$$

are also Majorana fermion operators. The following analysis for edge modes only involves two such operators.

The Hamiltonian *H*_*BW*_ accordingly can be rewritten as

13 $${H}_{BW}=\sum _{K}{h}_{BW}^{K},$$

14 $$\begin{array}{rcl}{h}_{BW}^{K} & = & -\frac{it}{2}\sum _{m\mathrm{=1}}^{M}[(1-{e}^{iK}){a}_{m,K}{b}_{m,-K}\\  &  & +{b}_{m+\mathrm{1,}-K}{a}_{m,K}-h.\,c.],\end{array}$$

which obeys

15 $$[{h}_{BW}^{K},{h}_{BW}^{{K}^{\text{'}}}]=\mathrm{0,}$$

i.e., *H*_*BW*_ has been block diagonalized. We note that for *K* = 0, we have

16 $${h}_{BW}^{0}=-\,\frac{it}{2}\sum _{m=1}^{M}({b}_{m+\mathrm{1,0}}{a}_{m\mathrm{,0}}-h.\,c.).$$

**Figure 1:**
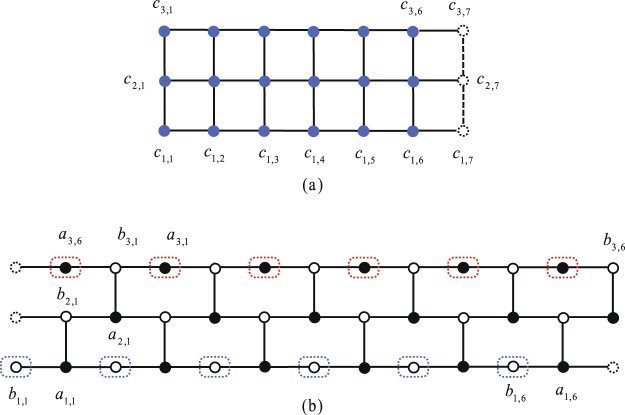
Schematic picture of the Kitaev model on a square lattice and its corresponding Majorana fermion system. (**a**) A 3 × 6 square lattice with periodic boundary condition in horizontal direction and open boundary in vertical direction. (**b**) The corresponding Majorana system which is a brick wall lattice with the same boundary conditions in lattice (**a**). Fermions *c*_*i,j*_ (blue circle) in (**a**) are decomposed into two Majorana fermions *a*_*i,j*_ and *b*_*i,j*_ (white and black circles, respectively) in (**b**). Majorana edge states for *a* and *b* are indicated by blue and red dotted circles, respectively, which are perfectly localized at the two edges of the cylinder.

Term *a*_*m*,0 *bm*,0_ disappears from the Hamiltonian, indicating the existence of an edge modes of Majorana fermions *a*_*m*,0_ and _*bm*,0_. It is a perfect edge mode with zero character decay length. The mechanism of the mode is the fact that, a honeycomb tube lattice with zigzag boundary is equivalent to a set of SSH chains^72^. The formation of such a state is the result of destructive interference at the edge. Figure 1 schematically illustrates the relation among the Kitaev model on a square lattice and the corresponding Majorana ferimonic model on a brick wall model, and the perfect edge modes, through a small size system^73,74^.

Actually, Hamiltonian ${h}_{BW}^{0}$ can be diagonalized by introducing *M* fermionic operators through

17 $${d}_{m}=\frac{1}{2}({a}_{m\mathrm{,0}}-i{b}_{m+\mathrm{1,0}}),{d}_{M}=\frac{1}{2}({a}_{\mathrm{1,0}}-i{b}_{M\mathrm{,0}}),$$

for *m* = 1, …, *M* − 1. Note operators that *d*_*m*_ (*m* ≠ *M*) combine the Majorana operators which derive from neighboring sites, while *d*_*M*_ combines the two ending Majorana operators. Using the above definition of *d*_*M*_, the Hamiltonian ${h}_{BW}^{0}$ can be written as the diagonal form

18 $${h}_{BW}^{0}=2t\sum _{m\mathrm{=1}}^{M-1}({d}_{m}^{\dagger }{d}_{m}-\frac{1}{2})+0\times {d}_{M}^{\dagger }{d}_{M}.$$

On the other hand, we note that

19 $$[{d}_{M},{h}_{BW}^{0}]=[{d}_{M},{H}_{BW}]=\mathrm{0,}$$

which means that *d*_*M*_ and ${d}_{M}^{\dagger }$ are the eigen operators of the Hamiltonian *H*_*BW*_ with zero energy. Operators *d*_*M*_ and ${d}_{M}^{\dagger }$ are refered as zero-energy mode operators, or edge-mode operators since only edge Majorana fermions *a*_1,0_ and *b*_*M*,0_ are involved. For an arbitrary eigenstate |Φ〉 of *H*_*BW*_ with eigenenergy *E*, i.e.,

20 $${H}_{BW}|{\rm{\Phi }}\rangle =E|{\rm{\Phi }}\rangle ,$$

state *d*_*M*_|Φ〉 $({d}_{M}^{\dagger }|{\rm{\Phi }}\rangle )$ is also an eigenstate of *H*_*BW*_ with the same eigenenergy *E*, if *d*_*M*_|Φ〉 ≠ 0 $({d}_{M}^{\dagger }|{\rm{\Phi }}\rangle \ne 0)$. In general, all the eigenstates of *H*_*BW*_ can be classified into two groups {|Φ_+_〉} and {|Φ_−_〉}, which are constructed as the forms

21 $$|{{\rm{\Phi }}}_{-}\rangle =\prod _{\{j\},j\ne M}{d}_{j}^{\dagger }|d-Vac\rangle ,|{{\rm{\Phi }}}_{+}\rangle ={d}_{M}^{\dagger }|{{\rm{\Phi }}}_{-}\rangle .$$

Here |*d* − *Vac*〉 is the normalized vacuum state of all fermion operators *d*_*j*_ (*j* ∈ [1, *M*])

22 $$|d-Vac\rangle ={\rm{\Lambda }}\prod _{j\mathrm{=1}}^{M-1}{d}_{j}|Vac\rangle ,$$

satisfying ${d}_{j}|d-Vac\rangle =0$, where Λ is the normalization factor. Obviously we have

23 $${d}_{M}|{{\rm{\Phi }}}_{-}\rangle =0.$$

We find that |Φ_−_〉 and |Φ_+_〉 possess the same eigen energy by acting with the commutation relation $[{d}_{M}^{\dagger },{H}_{BW}]=0$ on state |Φ_−_〉. Therefore, we conclude that all the eigenstates of *H*_*BW*_ is at least doubly degenerate and this degeneracy is associated with the existence of Majorana edge modes.

We are interested in the feature of edge-mode operator *d*_*M*_. It is easy to check that

24 $${d}_{M}=\frac{1}{2}({c}_{\mathrm{1,0}}^{\dagger }+{c}_{\mathrm{1,0}}-{c}_{M\mathrm{,0}}^{\dagger }+{c}_{M\mathrm{,0}}),$$

where

25 $${c}_{\mathrm{1,0}}=\frac{1}{\sqrt{N}}\sum _{n\mathrm{=1}}^{N}{c}_{\mathrm{1,}n},$$

26 $${c}_{M\mathrm{,0}}=\frac{1}{\sqrt{N}}\sum _{n\mathrm{=1}}^{N}{c}_{M,n},$$

are collective fermionic operators on the two edges of the cylinder. We note that edge-mode operator *d*_*M*_ is a linear combination of particle and hole operators of spinless fermion *c* on the edge with the identical amplitudes. To demonstrate the feature of the operator *d*_*M*_, we focus on two related states, vacuum state and excited of *d*_*M*_ particle (or hole and particle states). The vacuum state of fermion operator *d*_*M*_ can be constructed from *c* vacuum state as

27 $$\begin{array}{rcl}|M-Vac\rangle  & = & \sqrt{2}{d}_{M}|Vac\rangle \\  & = & \frac{1}{\sqrt{2}}({c}_{\mathrm{1,0}}^{\dagger }-{c}_{M\mathrm{,0}}^{\dagger })|Vac\rangle ,\end{array}$$

which satisfies ${d}_{M}|M-Vac\rangle =0$. Then the particle state is

28 $${d}_{M}^{\dagger }|M-Vac\rangle =\frac{1}{\sqrt{2}}(1+{c}_{M\mathrm{,0}}^{\dagger }{c}_{\mathrm{1,0}}^{\dagger })|Vac\rangle .$$

**Figure 2:**
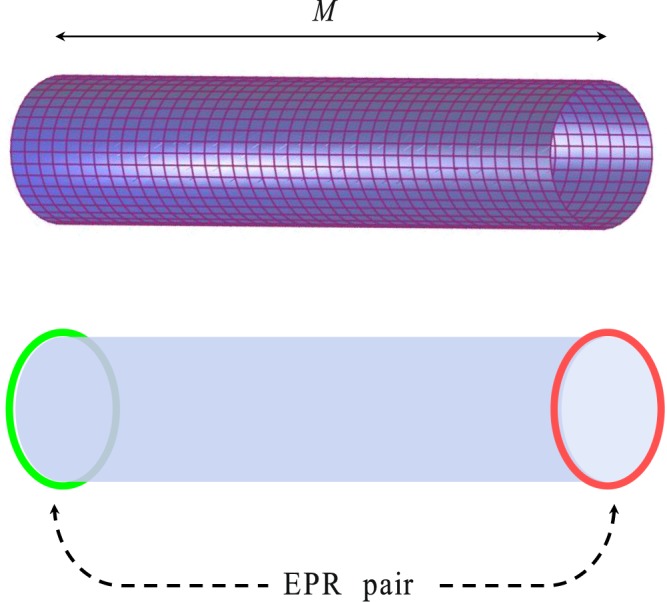
Schematics of the Kitaev model on a square lattice of cylindrical geometry with length *M* (upper panel). The Majorana zero-mode state corresponds to an EPR pair state of spinless fermions on the two edges of the cylinder (lower panel).

Remarkably, by the mappings of $|Vac\rangle \to {|\downarrow \rangle }_{M}{|\downarrow \rangle }_{1}$ and ${c}_{M\mathrm{,0}}^{\dagger }{c}_{\mathrm{1,0}}^{\dagger }|Vac\rangle \to {|\uparrow \rangle }_{M}{|\uparrow \rangle }_{1}$, which are based on the Jordan-Wigner transformation, we find that the edge particle state ${d}_{M}^{\dagger }|M-Vac\rangle$ is a maximally entangled state between two edges of the cylinder (see Fig. 2). On the other hand, if we take the mapping ${c}_{\mathrm{1,0}}^{\dagger }|Vac\rangle \to {|\uparrow \rangle }_{1}{|\downarrow \rangle }_{M}$ and ${c}_{M\mathrm{,0}}^{\dagger })|Vac\rangle \to {|\downarrow \rangle }_{1}{|\uparrow \rangle }_{M}$, we find that the edge hole state |*M* − *Vac*〉 is also a maximally entangled state. Although both states |*M* − *Vac*〉 and ${d}_{M}^{\dagger }|M-Vac\rangle$ are not eigenstates of *H*_*BW*_, the entanglement reflects the feature of the edge modes.

In short, a zero-energy mode is characterized by a conventional fermion operator, which is also referred as edge-mode operator. Any standard fermion operator has its own vacuum and particle states, or hole and particle states. We have shown that the corresponding hole and particle states for the edge-mode operators *d*_*M*_ and ${d}_{M}^{\dagger }$ are both EPR pair states in the spin representation. It reveals the non-locality of edge mode, through the particle and hole states are not eigenstates of the system.

For the eigenstate, the long-range correlation still exists. In the section Method, we show that the eigenstate |Φ_±_〉 can be regarded as entangled states between boson and fermion. It is expected that such a framework can be applied to more general cases.

## Summary

In this paper we have studied the edge modes of a finite size Kitaev model on a square lattice. The advantage of studying the finite system is that the obtained result can be demonstrated in synthetic lattice system. We studied the Majorana edge modes for the Kitaev model in a cylindrical geometry. The Majorana representation of the Hamiltonian turns out to be equivalent to a brick wall model under some conditions. The analytical solutions show that there exist perfect Majorana edge modes, which are in the strong localization limit. We provide a new way to analyze the excitation mechanisms in the framework of pseudospins for the edge modes. These modes, in contrast to the modes in Kitaev chain, can appear in small finite systems. This may provide a new venue for observing Majorana fermions in experiments.

## Method

### Pseudospin description

To get insight into the feature of the edge-mode related eigenstates in such a cylindrical Kitaev model, we introduce two types of pseudospin operators

29 $$\{\begin{array}{ccc}{s}^{x} & = & \frac{1}{2}({c}_{\mathrm{1,0}}^{\dagger }{c}_{M\mathrm{,0}}+{c}_{M\mathrm{,0}}^{\dagger }{c}_{\mathrm{1,0}})\\ {s}^{y} & = & \frac{1}{2i}({c}_{\mathrm{1,0}}^{\dagger }{c}_{M\mathrm{,0}}-{c}_{M\mathrm{,0}}^{\dagger }{c}_{\mathrm{1,0}})\\ {s}^{z} & = & \frac{1}{2}({c}_{\mathrm{1,0}}^{\dagger }{c}_{\mathrm{1,0}}-{c}_{M\mathrm{,0}}^{\dagger }{c}_{M\mathrm{,0}})\end{array},$$

and

30 $$\{\begin{array}{rcl}{\tau }^{x} & = & \frac{1}{2}({c}_{M\mathrm{,0}}^{\dagger }{c}_{\mathrm{1,0}}^{\dagger }+{c}_{\mathrm{1,0}}{c}_{M\mathrm{,0}})\\ {\tau }^{y} & = & \frac{1}{2i}({c}_{M\mathrm{,0}}^{\dagger }{c}_{\mathrm{1,0}}^{\dagger }-{c}_{\mathrm{1,0}}{c}_{M\mathrm{,0}})\\ {\tau }^{z} & = & \frac{1}{2}({c}_{M\mathrm{,0}}^{\dagger }{c}_{M\mathrm{,0}}+{c}_{\mathrm{1,0}}^{\dagger }{c}_{\mathrm{1,0}}-1)\end{array},$$

which satisfy the relations

31 $$[{s}^{\alpha },{s}^{\beta }]=i{\varepsilon }_{\alpha \beta \gamma }{s}^{\gamma },[{\tau }^{\alpha },{\tau }^{\beta }]=i{\varepsilon }_{\alpha \beta \gamma }{\tau }^{\gamma },$$

and

32 $$[{s}^{\alpha },{\tau }^{\beta }]=\mathrm{0,}$$

where $\alpha ,\beta ,\gamma =x,y,z$. Based on these relations the combination operator ${J}^{\alpha }={s}^{\alpha }+{\tau }^{\alpha }$ obeys the standard angular momentum relation

33 $$[{J}^{\alpha },{J}^{\beta }]=i{\varepsilon }_{\alpha \beta \gamma }{J}^{\gamma }.$$

We note that *J*^*x*^ is a conserved observable since $[{J}^{x},{H}_{BW}]=0$.

On the other hand, both operators *J*^*x*^ and *H*_*BW*_ are also invariant under a local particle-hole transformation ${\mathscr{P}}$, which is defined as

34 $${{\mathscr{P}}}^{-1}{c}_{M\mathrm{,0}}{\mathscr{P}}={c}_{M\mathrm{,0}}^{\dagger },{{\mathscr{P}}}^{-1}{c}_{M-\mathrm{1,0}}{\mathscr{P}}=-\,{c}_{M-\mathrm{1,0}}^{\dagger }.$$

The fact that, $[{J}^{x},{H}_{BW}]=[{\mathscr{P}},{H}_{BW}]=[{J}^{x},{\mathscr{P}}]=0$, tells us operators *J*^*x*^, ${\mathscr{P}}$ and *H*_*BW*_ share a common eigen vectors |Φ_±_〉. In fact, one pseudospin can be transformed to the other (and vice versa) by applying the transformation ${\mathscr{P}}$. Direct derivation shows that

35 $${{\mathscr{P}}}^{-1}{s}^{2}{\mathscr{P}}={\tau }^{2},$$

and

36 $${s}^{2}+{\tau }^{2}=3/\mathrm{4,}$$

which result in

37 $$\langle {{\rm{\Phi }}}_{\pm }|{s}^{2}|{{\rm{\Phi }}}_{\pm }\rangle =\langle {{\rm{\Phi }}}_{\pm }|{\tau }^{2}|{{\rm{\Phi }}}_{\pm }\rangle =3/8.$$

Together with

38 $${J}^{x}|{{\rm{\Phi }}}_{\pm }\rangle =\pm \,\frac{1}{2}|{{\rm{\Phi }}}_{\pm }\rangle ,$$

we find that state |Φ_±_〉 does not have definite values of *s* and *τ*. Unlike a standard spin operator which has its own vector space, operators {*s*^*α*^} and {*τ*^*β*^} share a common vector space.

Actually, for two edge sub-system, there are total four possible states which can be written down as

39 $$\begin{array}{rcl}|1\rangle  & = & |Vac\rangle ,|2\rangle ={c}_{\mathrm{1,0}}^{\dagger }|Vac\rangle ,\\ |3\rangle  & = & {c}_{M\mathrm{,0}}^{\dagger }|Vac\rangle ,|4\rangle ={c}_{\mathrm{1,0}}^{\dagger }{c}_{M\mathrm{,0}}^{\dagger }|Vac\rangle .\end{array}$$

We have the relations

40 $$\begin{array}{rcl}{s}^{z}|1\rangle  & = & {s}^{z}|4\rangle =\mathrm{0,}\\ {s}^{2}|1\rangle  & = & {s}^{2}|4\rangle =\mathrm{0,}\end{array}$$

and

41 $$\begin{array}{rcl}{s}^{z}|2\rangle  & = & \frac{1}{2}|2\rangle ,{s}^{z}|3\rangle =-\frac{1}{2}|3\rangle ,\\ {s}^{2}|2\rangle  & = & \frac{3}{4}|2\rangle ,{s}^{2}|3\rangle =\frac{3}{4}|3\rangle ,\end{array}$$

which mean that states |1〉 and |4〉 are spin state with *s* = 0, while |2〉 and |3〉 are spin states with *s* = 1/2. Simlarly, as for operator *τ*, we have

42 $$\begin{array}{rcl}{\tau }^{z}|2\rangle  & = & {\tau }^{z}|3\rangle =\mathrm{0,}\\ {\tau }^{2}|2\rangle  & = & {\tau }^{2}|3\rangle =\mathrm{0,}\end{array}$$

and

43 $$\begin{array}{rcl}{\tau }^{z}|4\rangle  & = & \frac{1}{2}|4\rangle ,{\tau }^{z}|1\rangle =-\frac{1}{2}|1\rangle ,\\ {\tau }^{2}|4\rangle  & = & \frac{3}{4}|4\rangle ,{\tau }^{2}|1\rangle =\frac{3}{4}|1\rangle ,\end{array}$$

which mean that states |2〉 and |3〉 are spin state with *τ* = 0, while |1〉 and |4〉 are spin state with *τ* = 1/2. Then if we regard operators {*s*^*α*^} and {*τ*^*β*^} as independent standard spin operators with *s*, *τ* = 0, 1/2, two degree of freedom in states |1〉, |2〉, |3〉, and |4〉 can be separated and written as direct product of two independent spin states

44 $$\begin{array}{rcl}|1\rangle  & = & {|0\rangle }_{s}{|\downarrow \rangle }_{\tau },|4\rangle ={|0\rangle }_{s}{|\uparrow \rangle }_{\tau },\\ |2\rangle  & = & {|\uparrow \rangle }_{s}{|0\rangle }_{\tau },|3\rangle ={|\downarrow \rangle }_{s}{|0\rangle }_{\tau },\end{array}$$

where

45 $$\begin{array}{rcl}{s}^{\alpha }{|0\rangle }_{s} & = & \mathrm{0,}\\ {s}^{z}{|\uparrow \rangle }_{s} & = & \frac{1}{2}{|\uparrow \rangle }_{s},{s}^{z}{|\downarrow \rangle }_{s}=-\,\frac{1}{2}{|\downarrow \rangle }_{s},\end{array}$$

and

46 $$\begin{array}{rcl}{\tau }^{\beta }{|0\rangle }_{\tau } & = & \mathrm{0,}\\ {\tau }^{z}{|\uparrow \rangle }_{\tau } & = & \frac{1}{2}{|\uparrow \rangle }_{\tau },{\tau }^{z}{|\downarrow \rangle }_{\tau }=-\,\frac{1}{2}{|\downarrow \rangle }_{\tau }.\end{array}$$

Obviously, this factorization of states is consistent with the Eqs from 40 to 43. In the spirit of this representation, one can construct equivalent states $|{\tilde{{\rm{\Phi }}}}_{\pm }\rangle$ to |Φ_±_〉 by regarding operators {*s*^*α*^} and {*τ*^*β*^} as standard spin operators with *s*, *τ* = 0, 1/2,

47 $$|{\tilde{{\rm{\Phi }}}}_{+}\rangle =\frac{1}{\sqrt{2}}({|0\rangle }_{s}{|\to \rangle }_{\tau }+{|\to \rangle }_{s}{|0\rangle }_{\tau }),$$

48 $$|{\tilde{{\rm{\Phi }}}}_{-}\rangle =\frac{1}{\sqrt{2}}({|0\rangle }_{s}{|\leftarrow \rangle }_{\tau }+{|\leftarrow \rangle }_{s}{|0\rangle }_{\tau }),$$

where

49 $${s}^{x}{|\to \rangle }_{s}=\frac{1}{2}{|\to \rangle }_{s},{s}^{x}{|\leftarrow \rangle }_{s}=-\,\frac{1}{2}{|\leftarrow \rangle }_{s},$$

50 $${\tau }^{x}{|\to \rangle }_{\tau }=\frac{1}{2}{|\to \rangle }_{\tau },{\tau }^{x}{|\leftarrow \rangle }_{\tau }=-\,\frac{1}{2}{|\leftarrow \rangle }_{\tau }.$$

We find that $|{\tilde{{\rm{\Phi }}}}_{\pm }\rangle$ has the same feature with |Φ_±_〉, i.e.,

51 $$\langle {\tilde{{\rm{\Phi }}}}_{\pm }|{s}^{2}|{\tilde{{\rm{\Phi }}}}_{\pm }\rangle =\langle {\tilde{{\rm{\Phi }}}}_{\pm }|{\tau }^{2}|{\tilde{{\rm{\Phi }}}}_{\pm }\rangle =\mathrm{3/8,}$$

52 $${J}^{x}|{\tilde{{\rm{\Phi }}}}_{\pm }\rangle =\pm \,\frac{1}{2}|{\tilde{{\rm{\Phi }}}}_{\pm }\rangle .$$

It indicates that eigenstate state |Φ_±_〉 originates from the couple of two types of excitations, boson and fermion. Particles *s* and *τ* have an internal degree of freedom, with quantum number 0 and 1/2, corresponding to bosonic and fermionic states. State |Φ_±_〉 can be regarded as the eigenstate of a boson-fermion coupling system. The state is maximally entangled between particles *s* and *τ* with the respect to the boson and fermion modes. Such an exotic feature is responsible to the existence of edge modes.
